# The immunometabolic function of VGLL3 and female-biased autoimmunity

**DOI:** 10.1097/IN9.0000000000000041

**Published:** 2024-05-08

**Authors:** Kameron Kennicott, Yun Liang

**Affiliations:** 1 Department of Physiology, Michigan State University, East Lansing, MI, USA; 2 Department of Pharmacology and Toxicology, Michigan State University, East Lansing, MI, USA

**Keywords:** autoimmunity, immunometabolism, nutrient sensing, sexual dimorphism

## Abstract

Autoimmune diseases exhibit a pronounced yet unexplained prevalence among women. Vestigial-like family member 3 (VGLL3), a female-biased factor that promotes autoimmunity, has recently been discovered to assist cells in sensing and adapting to nutritional stress. This role of VGLL3 may confer a selective advantage during the evolution of placental mammals. However, the excessive activation of the VGLL3-mediated energy-sensing pathway can trigger inflammatory cell death and the exposure of self-antigens, leading to the onset of autoimmunity. These observations have raised the intriguing perspective that nutrient sensing serves as a double-edged sword in immune regulation. Mechanistically, VGLL3 intersects with Hippo signaling and activates multiple downstream, immune-associated genes that play roles in metabolic regulation. Understanding the multifaceted roles of VGLL3 in nutrient sensing and immune modulation provides insight into the fundamental question of sexual dimorphism in immunometabolism and sheds light on potential therapeutic targets for autoimmune diseases.

## 1. Introduction

Autoimmune disease estimates place the United States between 25 and 50 million people affected, 75% of which being women ^[1,2]^. Its strong female bias is exemplified by diseases such as systemic lupus erythematosus, with a female-to-male ratio of 09:1; systemic sclerosis, with a female-to-male ratio of 11:1; and Sjögren’s syndrome (SS), with a female-to-male ratio of 14:1 ^[1]^. Research into the disparity between women and men in regard to these diseases has offered many leads. Sex hormones have been by far the most popular theory as they are easily distinguishable, potent, and have effects in a wide variety of organ systems ^[2]^. Sex chromosome-linked regulation comes in a close second, and epigenetic modifications, environmental triggers, gut microbiota, and developmental disparity bring up the rear ^[2]^. Currently, identified sex hormone and sex chromosome differences are inadequate to explain the pronounced female predominance of autoimmune diseases, suggesting the necessity for further studies to uncover new factors contributing to the female bias observed in these conditions.

## 2. VGLL3 as a novel factor in regulating sexual dimorphism in immunometabolism

Vestigial-like family member 3 (VGLL3) has emerged as a potential driver for autoimmune disease as well as its female bias ^[3–6]^. It is a putative transcriptional cofactor expressed at a higher level in females in normal human and mouse skin ^[3,6]^. The expression level of VGLL3 is further upregulated in autoimmune diseases, including systemic lupus erythematosus (SLE), SS, and rheumatoid arthritis (RA) ^[3]^. The upregulation of VGLL3 in SLE is seen in both female and male patients, suggesting its broad role in activating autoimmune disease pathways ^[3,6]^. This upregulation of VGLL3 is sex specific in that its basal level is higher in females. In other words, there is a lower barrier (ie, the difference between the basal and disease state) for VGLL3 level to reach a pathological level in females, explaining the higher risk in females. In human keratinocytes, reduction in VGLL3 levels downregulates genes that are hyperactivated in lupus skin ^[3]^. VGLL3 overexpression in mice recapitulates clinically important phenotypes of human cutaneous lupus and drives systemic autoimmunity with B-cell expansion, anti-dsDNA antibodies, and immune complexes in the skin and kidneys ^[6]^. The observations that VGLL3 is both elevated in normal female skin cells and activates inflammatory pathways key to lupus pathogenesis suggest its role in predisposing females to autoimmunity.

A novel mechanism underlying the pathogenesis of autoimmune diseases is the alteration in immunometabolism ^[4,7]^. In addition to classical functions in energy production and biosynthesis, distinct metabolic pathways can govern the phenotype and function of immune cell subtypes ^[8]^. Dysregulation of pathways such as pyruvate and lactate metabolism has been found in autoimmune diseases including multiple sclerosis and SS, as well as in signaling pathways such as the mammalian target of rapamycin-adenosine monophosphate (AMP) kinase pathway. These pathways are increasingly recognized as potential metabolic targets for SLE treatment ^[4,9,10]^.

Nutrient sensing plays a critical role in initiating metabolic processes that regulate cellular homeostasis, engaging anabolism and storage during food abundance and triggering mobilization of stored energy during scarcity ^[11]^. The significance of nutrient sensing is evident not just at the cellular level but also extends to the organismal and species levels throughout evolution. In the evolution of placental mammals, the necessity to nourish a developing embryo presented a considerable challenge to metabolism in maternal cells, which played a pivotal role in driving the development of nutrient-sensing pathways ^[12]^. Intriguingly, a recent study shows that increased levels of VGLL3 in females can help cells adapt to this metabolic challenge by sensing energy stress ^[5]^. Nutrient deficiency increases VGLL3 expression levels in keratinocytes, which subsequently leads to the upregulation of p53, a central player in the regulation of cellular metabolism ^[13]^. Nutrient sensing by the VGLL3-p53 axis supports the expression of interleukin (IL)-17C, an autocrine cytokine critical in innate immune function ^[14]^, to maintain basal defense in the epithelium. In contrast, nutrient sensing by VGLL3 does not induce the expression of cytokines that trigger systemic inflammation via long-range intercellular communication, which is known to be an energy-consuming process ^[5]^. Reduction in the level of VGLL3 results in defects in the activation of p53 and IL-17C in response to nutrient stress. Consequently, the VGLL3-p53 pathway reshapes the immune landscape to adapt to nutrient deficiency ^[5]^.

The role of VGLL3 in helping nonplacental female cells adapt to energy stress provides an evolutionary rationale for sexual dimorphism in immune regulation. However, hyperstimulation of the VGLL3 energy-sensing pathway can trigger autoimmune pathogenesis. Energy stress allows VGLL3 to be induced by interferon α (IFNα) in keratinocytes, causing inflammatory cell death and the exposure of self-antigens, which are known as hallmarks of SLE ^[5,15]^. This finding underscores the importance of maintaining metabolic homeostasis in preventing autoimmunity and identifies VGLL3 as a previously unknown factor in mediating sexual dimorphism in immunometabolism.

## 3. Regulation of VGLL3 expression and immunometabolism modulation

The induction of VGLL3 by nutritional stress suggests that VGLL3 may be regulated epigenetically and the regulation of VGLL3 expression is important for its immunometabolic roles. In a psoriasis model, 17-β-estradiol (E2) supplement in ovariectomized female mice has significantly increased its expression, which was reduced by the estrogen receptor beta (ER-β) antagonist PHTPP ^[16]^. E2 is known to regulate gene transcription via the modification of epigenetic marks on DNA and histone proteins including DNA methylation/demethylation and histone acetylation ^[17]^. As a growing body of work shows that E2 regulates immunometabolic pathways ^[18]^, it is of interest to test the hypothesis that E2 directs sex differences in immunometabolism through its epigenetic regulation of VGLL3 in future studies. Loss of heterozygosity of the VGLL3 locus is observed during tumorigenesis, a process tightly associated with immunometabolic rewiring, suggesting that epigenetic mechanisms may affect VGLL3 expression ^[19,20]^. Furthermore, histone acetylase inhibitors have been shown to induce VGLL3 expression ^[21]^. In addition, methylation of CpG sites has been identified at the VGLL3 promoter in humans and the methylation level correlates with VGLL3 expression changes in blood cells ^[22]^. As VGLL3 levels are upregulated in a number of autoimmune diseases ^[3,5]^, it will be of future interest to determine whether changes in metabolic states lead to hypomethylation in *VGLL3* to impact disease pathogenesis.

## 4. VGLL3 and Hippo – interaction of nutrient-sensing pathways

Initially identified as a member of the Vestigial family, VGLL3 is hypothesized to bind to the TEA-domain-containing transcription factors (TEADs) through its TONDU domain, the domain in the human protein TONDU that is homologous to the domain of the Drosophila protein Vestigial required for interaction with Scalloped, and act as TEAD cofactors in mediating its cellular functions ^[23]^. TEAD regulates transcription with its cofactors Yes-associated protein (YAP) and transcriptional coactivator with PDZ-binding motif (TAZ), which are downstream effectors of the Hippo pathway indicating the “off” status of Hippo. When Hippo is “on,” either mitogen-activated protein kinase 4 (MAPK4) or the combination of mammalian sterile 20-like kinases 1/2 and its associated scaffold protein Salvador family with WW domain containing protein 1 phosphorylate large tumor suppressor kinases 1/2 (LATS1/2) and its scaffold MOB kinase activator 1, leading to the subsequent phosphorylation of YAP/TAZ. Phosphorylated YAP/TAZ is inactivated and is prevented from translocating into the nucleus and activating downstream genes ^[24]^.

Of note, the Hippo pathway is an evolutionarily conserved stress sensor. Hippo responds to a number of stress signals including hypoxia, heat, osmotic, and energy stresses to regulate cell growth, polarity, and differentiation. For example, hypoxia triggers downregulation of YAP phosphorylation but upregulation of TAZ phosphorylation in ovarian cancer cells, suggesting that limited oxygen supply has a complex effect on Hippo activation ^[25]^. Heat stress can activate YAP/TAZ to induce the heat shock transcriptome ^[26]^, while osmotic stress can inhibit YAP/TAZ function ^[27]^. Energy stress leads to AMP kinase-dependent LATS activation and subsequent phosphorylation of YAP, which modulates cellular events including cell growth, proliferation, apoptosis, and differentiation ^[28,29]^.

Consistent with its critical role in regulating cell proliferation and differentiation, Hippo is recognized as a tumor suppressor pathway ^[24]^. In tumor cells, *VGLL3* expression leads to the inactivation of YAP/TAZ and the combination of VGLL3 with TEAD expression promotes expression of Hippo pathway genes ^[30]^. These results are consistent with the proposed model in which VGLL3 competes with YAP/TAZ for TEAD to regulate transcription, but a biochemical study directly testing this hypothesis is needed and the model may be cell-type and stimuli dependent. VGLL3 has been found to modulate glutamine metabolism through regulating the expression of glycinamide ribonucleotide formyltransferase (GART), which encodes a trifunctional enzyme that catalyzes de novo purine synthesis from glutamine in cancer cells ^[31]^. Immunologically, VGLL3 has been shown to be associated with the abundance of macrophages and dendritic cells in tumor infiltrates ^[32]^. Collectively, these lines of evidence indicate a dynamic interaction between VGLL3 and the Hippo pathway that may be associated with the immunometabolic dysregulation in tumorigenesis, which is a sexually dimorphic process ^[33]^.

With classically defined functions in organ size regulation and proliferative control, in immune contexts, the Hippo pathway can regulate lymphocyte development, trafficking, and survival as well as regulatory and effector T-cell differentiation. Further, recent findings have associated loss of function Hippo variants in humans and knockouts in mice with female-biased autoimmune disorders ^[34–36]^. In RA, a female-biased autoimmune disease, VGLL3 has been shown to modulate the expression of Hippo “off” pathway molecules WWTR1 (TAZ) and AMOTL2 in patient-derived fibroblast-like synoviocytes ^[37]^. In turn, these Hippo pathway molecules mediate the regulation of interferon regulatory factor 3 (IRF3) and IFN-β1 by VGLL3, suggesting that VGLL3 receives signal from the Hippo stress-sensing pathway to activate IFN responses, a key immunological pathway in autoimmune disease ^[37]^. Recent studies indicate that IFN response in human skin is dependent on HERC6, an IFN-induced E3 ubiquitin ligase that modulates the intersection between Hippo and antiviral pathway through LATS2 and IRF3 regulator TBK1 in a VGLL3-dependent manner ^[38]^. Furthermore, VGLL3 regulates the expression of c-Jun, which can directly activate the transcription of YAP1, a major readout of the Hippo pathway ^[39,40]^. As a critical regulator of glycose and glutamine metabolism, c-Jun is known to activate inflammatory pathways and its regulation by VGLL3 is dependent on the canonical Hippo pathway, suggesting again the presence of a feedback loop between the two pathways ^[40–43]^. Therefore, there is a growing interest in understanding the potential crosstalk between the VGLL3 and Hippo pathways in regulating sexual dimorphism in autoimmunity, exemplified by studies showing that VGLL3 drives IFN-β1 expression in fibroblast-like synoviocytes of RA patients by inhibiting TAZ1 expression ^[37]^.

## 5. VGLL3-regulated effectors in immunometabolism and autoimmunity

Under nutritional stress, VGLL3 is known to increase β-defensins in skin, primarily β-defensin 3 (DEFB3) ^[5]^. While defensins are typically thought to be helpful, a growing body of literature supports microbial exploitation of these normally antimicrobial peptides ^[44]^. Likewise, in squamous cell carcinomas of the head and neck, β-defensin 3 promotes the induction of master regulator NFκB induction leading to immune activation, chemokine signaling through CCR7, and antiapoptotic effects ^[45]^. In cancer this is detrimental because it may allow the cancer to escape apoptosis, attract tumor-associated macrophages, and increase metastasis, but what about autoimmunity? A chronic inflammatory state caused by overexpression of proinflammatory cytokines via β-defensin 3 induction of NFκB may set the stage for the breakage of tolerance in skin. Importantly, NFκB itself is known for a central role in coordinating inflammatory and metabolic programs, being regulated by a great number of metabolic factors such as glutamate dehydrogenase 1, hexokinase 1, and TP53-induced glycolysis regulatory phosphatase as well as regulating multiple metabolic genes such as *Pfkm*, *Fads2*, and *Fasn* ^[46]^.

B-cell activating factor (BAFF) is an immunological target positively regulated by VGLL3 expression in keratinocytes, B-cells, and monocytes ^[3]^. BAFF is a critical B-cell survival factor without which maturation of B-cells does not occur ^[47]^. BAFF deficiency results in a reduced number of peripheral B-cells and diminished ability to mount robust immune responses, and conversely, BAFF overexpression has been associated with human and murine autoimmunity ^[48,49]^. BAFF functions as a proinflammatory cytokine by binding either its eponymous receptor BAFFR as a homotrimer or TACI and BCMA as a 60-mer. Binding to any of these receptors activates NFκB pathways, most potently through BAFFR and the NFκB2 alternative pathway in B-cells, causing spontaneous breakage of tolerance and lupus-associated phenotypes ^[50]^. In TLR7 and Sle1 transgenic mouse models of lupus, BAFF from monocytes is required for activation, maturation, and autoantibody production in pathogenic B-cells ^[51]^. The immunometabolic function of BAFF is shown by its initiation of signaling cascades involving PI3K/AKT/mammalian target of rapamycin, which are known to regulate glycolysis, protein synthesis, lipid synthesis, and oxidative stress ^[52]^. BAFF-induced activation of glycolysis and oxidative phosphorylation is thought to provide the necessary molecular building blocks and energy to support cell mass generation. Therefore, the heightened expression of BAFF due to VGLL3 may predispose women to autoimmune disorders with directly or indirectly impacted metabolic reprogramming.

Type 1 IFNs are known for their antiviral, antitumor, and immunomodulatory effects and have been shown to be a key driver in female-biased autoimmune diseases including SLE, SS, and RA ^[53]^. The upregulation of IFN-induced genes, including *BAFF* and *IL7*, which stimulate B- and T-cell proliferation and differentiation, by VGLL3 in multiple cell types suggests a direct mechanism by which VGLL3 promotes autoimmune pathogenesis ^[3]^. The immunometabolic role of type 1 IFNs is indicated by studies demonstrating IFN-induced alterations in core metabolism, including fatty acid oxidation and oxidative phosphorylation, in plasmacytoid dendritic cells and nonhematopoietic cells ^[54]^. IFNβ-IFN-α/β receptor signaling associates with decreased glycolysis and mitochondrial dysfunction and stress in inflammatory macrophages ^[55]^. IFN-stimulated genes including *CH25H*, *SAT1*, *IDO1*, and *SAMHD1* are shown to modulate specific metabolic events during IFN-induced immune responses ^[56]^. Upstream, it was recently discovered that lactate induces acinar epithelial cell atrophy, a hallmark of SS, alongside the production of multiple proinflammatory cytokines and type 1 IFN pathway activation ^[9]^.

IL-1ɑ is a potent inflammatory cytokine whose deregulated signaling causes severe acute or chronic inflammation ^[57]^. It is already expressed under homeostatic conditions, immediately bioavailable, and its receptor (IL1R) is constitutively expressed in many cell types. Upon cellular injury, it begins an inflammatory signaling cascade through IL1R-induction of MAPK and NFkB, leading to further proinflammatory cytokine expression of tumor necrosis factor alpha (TNFɑ), IL-6, and cyclooxygenase-2 (COX-2) that in turn positively feedback into IL-1ɑ production ^[57]^. If the cell undergoes apoptosis, IL-1ɑ is sequestered into the nucleus and phagocytosed, preventing the initiation of an inflammatory response ^[57]^. VGLL3 has been shown to mediate IL-1ɑ maturation and secretion, which is not a typical outcome for the cytokine ^[58,59]^. This is likely due to inflammasome signaling leading to membrane pore formation and pyroptosis ^[57]^. This would suggest that women would produce and secrete higher levels of IL-1ɑ due to higher VGLL3 expression, though sex hormones may also play a role ^[60]^. Therefore, women may be primed for faster and larger immune responses relative to the insult compared with men due to a VGLL3-mediated increase in homeostatic IL-1ɑ levels. The metabolic role of the IL-1 pathway is shown by the IL-1 receptor antagonist–deficient (IL-1Ra^−/−^) mice, which exhibit impaired body fat accumulation without abnormalities in feeding behavior or expression of hypothalamic factors ^[61]^. An unconventional IL-1R-MyD88-IRK2-prohibitin/optic atrophy protein 1 signaling axis has been found to suppress oxidative metabolism in adipocytes ^[62]^. Considering the suggested link between adipocyte-associated chronic inflammation and autoimmunity ^[63]^, there is significant interest in studying the immunometabolic role of VGLL3-IL-1ɑ signaling in SLE and additional autoimmune diseases.

## 6. Conclusions

**Figure 1.**
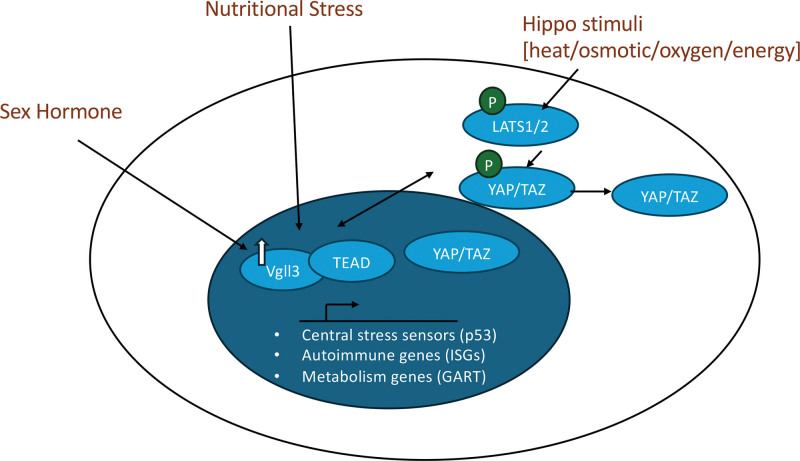
**Outline of the role of VGLL3 in autoimmunity.** VGLL3 senses nutritional stress and increases in expression level to activate central stress sensors including p53 and c-Jun. VGLL3 forms a transcriptional complex with TEAD, which is also known to interact with YAP/TAZ, effectors of the Hippo pathway sensing changes in heat, osmotic, oxygen, and energy levels. VGLL3 regulates Hippo pathway components and vice versa, potentially forming a feedback mechanism. Unattenuated VGLL3 upregulation leads to inflammatory cell death and exposure of autoantigens. Additionally, VGLL3 affects the expression of metabolism genes such as *GART* and VGLL3 upregulates autoimmune genes such as *ISGs*, *NFκB*, *BAFF*, and *IL-1*ɑ that are known to impact various metabolic pathways. BAFF, B-cell activating factor; ISGs, Interferon-stimulated genes; TAZ, transcriptional coactivator with PDZ-binding motif; TEAD, TEA-domain-containing transcription factors; YAP, Yes-associated protein.

VGLL3 is a female-biased factor that helps cells sense and adapt to nutritional stress. This function of VGLL3 may provide a selective advantage during the evolution of placental mammals. However, hyperactivation of the VGLL3-mediated energy-sensing pathway can lead to inflammatory cell death and the development of autoimmunity. Loss in VGLL3 results in dysregulation of central immunometabolic pathways including p53 and Hippo, suggesting that VGLL3 expression plays a key role in impacting responses to nutrient changes beyond being a consequence. Mechanistically, VGLL3 intersects with Hippo signaling and activates multiple downstream, immune-associated genes that play roles in metabolic regulation (Figure 1). Combined, VGLL3’s regulation of immune and metabolic networks supports the idea that it regulates sexually dimorphic immune regulation and contributes to the increase in risk of autoimmunity for women.

## Conflicts of interest

The authors declare no conflict of interest.

## Funding

Y.L. has received research support from the NIH National Institute of Arthritis and Musculoskeletal and Skin Diseases grant K01 AR073340, R01 AR078781 and National Institute of Dental and Craniofacial Research grant R21 DE031765.
